# A Scale-Adaptive Matching Algorithm for Underwater Acoustic and Optical Images

**DOI:** 10.3390/s20154226

**Published:** 2020-07-29

**Authors:** Jun Liu, Benyuan Li, Wenxue Guan, Shenghua Gong, Jiaxin Liu, Junhong Cui

**Affiliations:** 1 College of Computer Science and Technology, Jilin University, Changchun 130012, China; liujun1509@jlu.edu.cn (J.L.); liby18@mails.jlu.edu.cn (B.L.); gongsh18@mails.jlu.edu.cn (S.G.); liujiaxin17@mails.jlu.edu.cn (J.L.); junhong_cui@jlu.edu.cn (J.C.); 2 College of Electronic Information Engineering, Beihang University, Beijing 100191, China

**Keywords:** underwater detection, image matching, correlation filter, image enhancement

## Abstract

Underwater acoustic and optical data fusion has been developed in recent decades. Matching of underwater acoustic and optical images is a fundamental and critical problem in underwater exploration because it usually acts as the key step in many applications, such as target detection, ocean observation, and joint positioning. In this study, a method of matching the same underwater object in acoustic and optical images was designed, consisting of two steps. First, an enhancement step is used to enhance the images and ensure the accuracy of the matching results based on iterative processing and estimate similarity. The acoustic and optical images are first pre-processed with the aim of eliminating the influence of contrast degradation, contour blur, and image noise. A method for image enhancement was designed based on iterative processing. In addition, a new similarity estimation method for acoustic and optical images is also proposed to provide the enhancement effect. Second, a matching step is used to accurately find the corresponding object in the acoustic images that appears in the underwater optical images. In the matching process, a correlation filter is applied to determine the correlation for matching between images. Due to the differences of angle and imaging principle between underwater optical and acoustic images, there may be major differences of size between two images of the same object. In order to eliminate the effect of these differences, we introduce the Gaussian scale-space, which is fused with multi-scale detection to determine the matching results. Therefore, the algorithm is insensitive to scale differences. Extensive experiments demonstrate the effectiveness and accuracy of our proposed method in matching acoustic and optical images.

## 1. Introduction

In recent years, the research of underwater exploration using either acoustic imaging or optical imaging alone has made significant contributions to applications of underwater target detection, underwater archaeology, seabed resource exploration, biological research, underwater environment monitoring and other fields [1,2,3,4,5]. These two image exploration methods have their own advantages, but they also have some constraints. The information obtained from underwater optical imaging has the advantages of high imaging resolution and high information content, and is more intuitive. However, the absorption and scattering effects caused by water are two major constraints of underwater optical imaging. Due to the absorption of water, the imaging beam is unable to reach the imaging plane beyond a certain distance, which seriously limits the range of underwater optical imaging. In addition, scattering can change the distribution of light energy in space and time, produces high levels of noise, affects the imaging signal-to-noise ratio, and can even prevent imaging when backscattering is significant. In contrast to a light wave, an acoustic wave, which is an elastic wave, has the advantages of small loss and long propagation distance. It can travel tens of kilometers or more in water. It is an ideal detection signal in terms of distance. However, according to the basic characteristics of marine acoustics, the propagation speed of acoustic waves in seawater is seriously affected by environmental factors such as temperature, salinity, and water pressure, which has a great impact on sonar detection. This directly changes the propagation track of acoustic waves in seawater: when the sound velocity changes to a positive gradient, the sound line emitted by an underwater sound source bends toward the sea surface; when the sound velocity changes to a negative gradient, the sound line moves toward the seabed. Due to the heterogeneity and variability of marine media, the distribution of sound velocity is very complex. Therefore, the propagation law of acoustic waves in the ocean not only depends on the boundary conditions of the ocean, such as the temperature, salt distribution, and composition changes of the sea water, but is also restricted by marine dynamic factors and space–time changes. Thus, sonar imaging sometimes causes a large deviation underwater. In addition, there are complex noises in the marine environment, which not only include the sound generated by the movement of the marine medium itself, but also the sound from most marine organisms, which results in significant interference in the image. This interference covers the entire acoustic frequency band; furthermore, the waveforms range from a pulse wave to a sine wave and their distribution is irregular. Due to the long-distance propagation ability of acoustic waves in the ocean, all types of noise interfere with sonar detection, which results in low sonar image quality and makes it difficult to accurately displaying the details of underwater exploration targets. At present, sonar equipment mainly comprises a transducer array, transmitter, receiver, transceiver conversion device, and terminal display equipment. The transmitter generates a certain form of modulated electrical signal, which is sent to the transducer array by the transceiver conversion device. This is then converted into acoustic energy to radiate into water. At the same time, part of the energy of the signal is coupled to the receiver as the timing start (distance zero) signal. When the acoustic signal meets the target in the course of transmission, part of the acoustic energy is reflected back to the transducer and converted into an electrical signal, which is sent to the receiver for amplification by the transceiver conversion device, and then sent to the terminal display device for observation. Thus, the sonar diagram is finally formed. Therefore, one of the remaining challenges is the integration of acoustic and optical data. This integration is the preliminary step in the latest research into techniques such as joint positioning, image fusion, and target recognition. For example, in the target location of an object captured by optical images, based on the matching of the optical and sonar images, the underwater optical images and the sonar measurement results can be fused, and the target position can be located based on the TOA (Time of Arrival) information and the AUV’s (Autonomous Underwater Vehicle) position information of the sonar. In addition, by matching the target in the optical and sonar images, the details of the target object in the acoustic images can be further explored using the information (color, contour, etc.) of the optical images.

In this study, a fast image matching method based on a correlation filter [6,7] and Gaussian scale-space [8] was designed for multi-scale image matching. In the process of target matching between optical images and sonar data (acoustic images), underwater optical images and sonar measurement data of the same scene are significantly different in terms of image definition, color, dynamic range, signal-to-noise ratio, and structural similarity. To ensure the optimal results of the matching, enhancement is needed for raw images. Image enhancement mainly aims to solve the problems of image color distortion, contour blur, detail loss, and noise suppression. Using image similarity evaluation and enhancement with an iterative algorithm designed in this work, we can obtain enhanced images with obvious features from the lower-quality original images. These enhanced images are suitable for subsequent matching. At present, no unique algorithm exists for the fusion of underwater optical and acoustic images in existing object detection methods [9,10]. We designed a special matching algorithm based on the Gaussian scale-space and correlation filter to perform multi-scale matching of the underwater images to be detected. Considering the characteristics of the underwater images with fuzzy edges and greater noise, we used the region-based minimum output sum of squared error filter to perform the matching. The filter is based on the cross-correlation between acoustic and optical images to match the images, so is not sensitive to the effects of edge blur, image incoherence, and slight deformation of underwater images. Moreover, it has a high tolerance to noise. In order to eliminate the influence of scale transformation caused by different camera angles, we introduce the Gaussian scale-space to build multi-scale images. Finally, by combining the multi-scale matching results of the Gaussian scale-space, accurate matching results are obtained. The experimental results show that the algorithm can quickly and accurately find the corresponding object in the acoustic images for the specified object in the underwater optical images.

## 2. Related Work

To solve the problems of acoustic and optical data fusion [11,12], a matching algorithm for image data of different sensor sources is required. As a classic topic, numerous image matching systems have been proposed during the past decade [13,14,15]. Image matching algorithms include region-based and feature-based image matching algorithms The region-based matching algorithm is based on the correlation within a group of comparative image sets, whereas the feature-based matching algorithm is based on feature information such as contour lines of objects.

Mean absolute differences (MAD) is a matching algorithm proposed by Leese in 1971. By traversing the entire image, the algorithm calculates the average absolute difference between the target template and all of the selected subgraphs. The smaller the average absolute difference, the higher the similarity. This matching method requires high imaging quality, is easily affected by noise, and has several costly computations. In 1972, Barnea and Slaverman proposed the sequential similarity detection algorithm (SSDA algorithm) [16], which is an improvement of the MAD method. By setting the threshold, all of the pixels are avoided, and the computing speed is significantly increased. Essentially, these algorithms are based on the template matching of the gray level, which leads to the problems of oversensitivity to the change of the image’s gray level. Because of the complex imaging environment, underwater images have the characteristics of poor imaging quality and a significant amount of noise. In addition, due to the attenuation and scattering of light in the water, the scattering rate of low-frequency light (red and yellow light) is low, whereas that of high-frequency light (blue and green) is high. This leads to color distortion of underwater optical images, which generally show a green or blue bias. These characteristics greatly affect the accuracy of the algorithm applied to acoustic and optical image matching and limit its practical application in underwater environments. Medioni proposed a matching method based on linear features in 1984 [17], and Li proposed a matching method based on contours in 1995. Matching methods based on lines or edges have a strong anti-interference ability for the change of gray level, but because the matching is based on edge information, the matching effect will also be affected by the quality of edge extraction. In underwater environments, the suspended particles in turbid water lead to light scattering, which blurs the picture with the effect of smog. This leads to the blurring of the image outline and the loss of details. To ensure the application of these algorithms in underwater acoustic and optical image matching, additional edge enhancement and extraction must be carried out. Moreover, the influence of sound and shadow should be considered. The sound shadow phenomenon leads to the generation of a sound shadow area, which is mainly due to obstacles or the refraction effect, and cannot be reached by a sound wave. In the sound shadow area, only reverberation and partial reflection sounds can be received. The sound shadow area seriously affects the feature extraction of the target object, and effective edge information cannot be obtained. In addition, because acoustic and optical images are captured by different sensors, it is difficult to guarantee the image angles are the same in complex underwater environments. Such methods are sensitive to changes in the angle of shots and the deformation of images should thus be considered. Correlation filters were originally applied in signal processing to describe the correlation between two signals. In 2010, David S. Bolme introduced the correlation filter into target tracking for the first time and proposed a method using calculation of the correlation [18]. Correlation filtering is an image matching and tracking method based on the correlation found in a set of comparative images. The filter is based on the cross-correlation of underwater images and is insensitive to edge blurring, incoherence, and slight deformation of underwater images, and has a high tolerance for noise. The filter is robust to noise and does not depend on statistical properties. It can also adapt to the scene of underwater images better with multi noise and object deformation. However, the scale change of the target is not considered in the algorithm. In the process of underwater acoustic and optical image matching, the matching accuracy will be reduced if the size of the target is different due to the change of the angle.

At present, no object detection algorithm exists based on the fusion of underwater optical and sonar images. Based on correlation filtering, in this study we designed a matching method for acoustic and optical images, and introduced Gaussian scale-space to build multi-scale images to eliminate the influence of scale difference. In addition, in the process of matching optical images and a sonar data (acoustic images) target, the underwater optical images and sonar measurement data of the same scene are significantly different in terms of image definition, signal-to-noise ratio, and structural similarity. Thus, in the subsequent matching step, the original images must be preprocessed and enhanced to ensure the accuracy of image matching. Image enhancement is mainly aimed at the problems of color distortion, contour blur, detail loss, and noise suppression of underwater images. Using image similarity evaluation and the iterative enhancement algorithm we designed, two types of images with relatively less noise, sufficient detailed information, and high similarity are obtained. The experimental results of dozens of typical scenes show that our method can perform acoustic and optical image matching more accurately.

## 3. Proposed Method

### 3.1. System Overview

**Figure 1:**
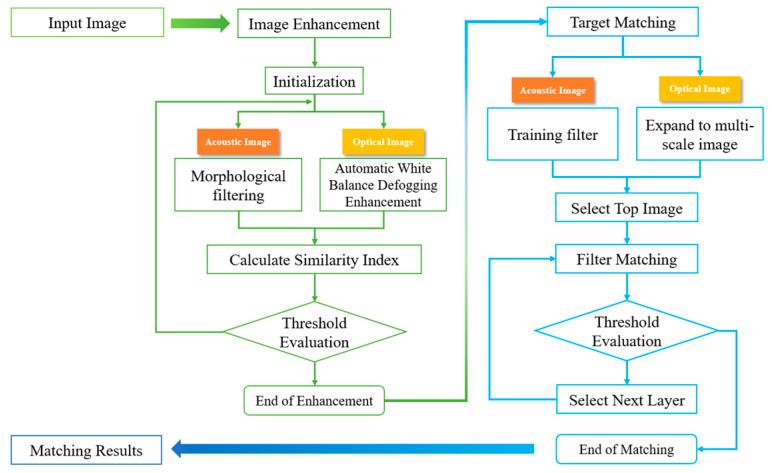
Overall framework of matching algorithm.

The overall system consists of two parts: image preprocessing and image matching. The optical image preprocessing is based on gray world automatic white balance and dark channel priority [19,20]. For acoustic images, morphological filtering is used [21]. Based on the previous research discussed, we performed correlation-based image matching. The image matching algorithm includes two processes: training and matching. The correlation filter is trained by extracting the spatial features of the images. By expanding the target image to a series of multi-scale images, the filter is detected in each layer. The detection of the multi-scale images is then carried out [22]. Finally, through the fusion of the multi-scale detection results, accurate matching results are obtained. The specific process is shown in Figure 1.

### 3.2. Image Pre-Processing

Two problems need to be solved in underwater optical image enhancement. Firstly, due to the attenuation and scattering of light in water, the scattering rate of low-frequency light (red and yellow light) is low, whereas that of high-frequency light (blue and green light) is high. This leads to the color distortion of underwater optical images. The images generally show a green or blue bias. Secondly, suspended particles in the turbid water cause light scattering, which blurs the picture and causes the picture to suffer from the effect of smog. This leads to the loss of image detail, blurring of contours, reduction of contrast, and a decrease of the signal-to-noise ratio [23]. The quality problem of underwater acoustic images is manifested by the presence of more noise, which affects most of the extracted features, thereby reducing the matching success rate.

We used color consistency enhancement to address the problem of color distortion in optical images and a defog algorithm to deal with the problems of image blur and detail loss. Based on the full scattering and uniform environment light characteristics of underwater optical images, color distortion is often solved using a white balance algorithm of uniform light. Considering the complex and varied underwater environment, the stability of the algorithm in extreme water quality, and the calculation speed, the gray world automatic white balance can effectively avoid color distortion by estimating the color deviation of the whole image. For underwater pictures, this has a wider range of use, can be applied in extreme cases (e.g., when the water quality is overly turbid, leading to blurring of the object contour and loss of color information due to the lack of an object), and has a faster operating speed than other methods [24,25]. The gray world automatic white balance algorithm is based on the gray-scale world hypothesis, which is used as a priori knowledge and applied to the images to be processed to eliminate the influence of ambient light from the images and obtain the original scene images. Through the analysis of ten typical underwater images, it was found that using the gray world automatic white balance algorithm can largely eliminate the phenomenon caused by scattering in which high-frequency light is significantly higher than low-frequency light.

An image defogging algorithm is often used to solve the problems of blur and detail loss of underwater images. At present, the commonly used image defogging algorithms include maximum contrast method, color attenuating prior method, and a dark channel prior algorithm [26,27,28]. Based on the fact that underwater scenes are characterized by multiple sources of noise and serious blur, and considering the need to preserve the object contour in the process of defogging enhancement, the dark channel priority algorithm has the advantages of effectively suppressing the noise and halo phenomenon, and quickly completing defog processing. After the image data is processed by the dark channel prior algorithm, the details of the target object are enhanced, and the definition of the contour edge is greatly improved.

**Figure 2:**
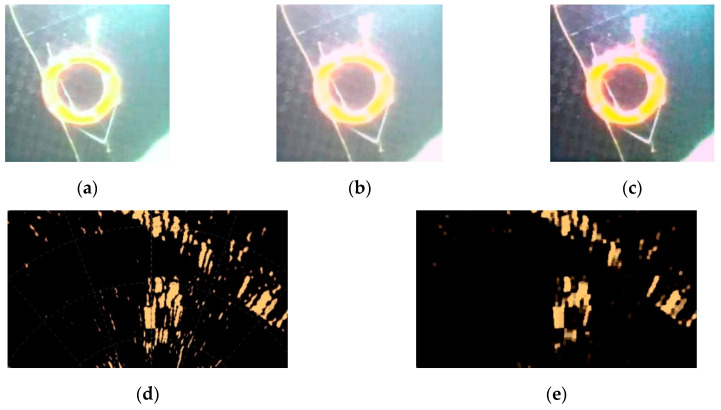
**(a**) The original optical image; (**b**) the image after color consistency enhancement; (**c**) the image after defogging; (**d**) the original acoustic image; (**e**) the image after noise reduction.

Because sonar images are significantly affected by noise, low definition, and unclear edge contours, underwater acoustic images need to be repaired and smoothed. Therefore, a morphological filtering algorithm should be used to suppress noise without loss of image detail [29]. For preserving image details and adaptive processing, morphological filters are more practicable than other methods. This kind of filter can reduce the loss of image detail and maintains the geometric features of the images as much as possible. Figure 2 shows the effect of some enhancement techniques.

### 3.3. Iterative Enhancement Based on Matching Degree

Based on the image enhancement algorithms discussed above, we utilized an iterative enhancement Algorithm 1 using ascending gradient calculation and gradually adjusted its parameters to make the two images consistent in terms of structural similarity and peak signal-to-noise ratio. In addition, we used an image matching measurement method to evaluate the similarity between underwater acoustic and optical images, and used the similarity index to determine the adjustment rate of the enhancement algorithm parameters. The procedure is as follows:
**Algorithm 1** Iterative Enhancement**Input:** Original image; Initial values of parameters; **Output:** Enhanced image with high similarity **1: procedure** Iterative Enhancement **2:** initial θ, α, e; **3: repeat:** **4:** optimage←AWB-Defogging (optimage, θ, α); **5:** acoimage←Morphology (acoimage, e); **6:** CalGradient (θ, α, e); **7:** SI ←CalSI(); **8: until** (SI > Threshold); **9: end procedure**

1. Determine the initial parameters of the white balance, defog, and morphological filtering algorithm, including the color deviation retention parameter θ of the gray world automatic white balance algorithm, the ambient light retention parameter α in the dark channel priority algorithm, and the morphological filtering constraint E.
2. The existing parameters are used to enhance the underwater acoustic images and underwater optical images. For optical images, the gray world based automatic white balance algorithm and dark channel priority defogging algorithm are used. Morphological filtering is used to enhance the acoustic images.
3. For the enhanced images in the second step, the peak signal-to-noise ratio (PSNR) and structural similarity (SSIM) are used to measure the similarity of the two images, and weighted fusion is carried out according to the influence of each index on similarity to obtain the final similarity index (SI). If the similarity index is higher than the specified threshold, the algorithm ends. Otherwise, it continues with step 4. The specific SI values are calculated as follows:
  (1) $$\mathrm{SI}=W_{1}\cdot \mathrm{PSNR}+W_{2}\cdot K\cdot \mathrm{SSIM}$$
  where w_1_ and w_2_ are the weight coefficients valued at 0.3 and 0.7, respectively, and K is the coefficient value of measurement difference, which is valued at 15. The experimental results show that when the SI threshold value is taken 12, the overall image enhancement effect is better.
4. Determine the parameters of the enhancement algorithm in the next iteration. The similarity index has a functional relationship with the color deviation retention parameter θ of the gray world automatic white balance algorithm, the ambient light retention parameter α in the dark channel priority algorithm, and the morphological filter constraint e: SI = J (θ, α, e). We used the gradient ascent method to obtain the parameter difference between the next iteration and the current iteration, and thus obtain the parameters of the next iteration. The algorithm then returns to the second step to continue the iteration. Through the iterative enhancement of the two images, the problems of color distortion, contour blur, detail loss, and noise suppression are addressed. In addition, the two images tend to be consistent in terms of structural similarity and signal-to-noise ratio.

### 3.4. Image Matching Based on Image Spatial Features

Using image preprocessing and enhancement, the noise in the images is significantly reduced, and the target information is fully exposed. For the enhanced images, we match the acoustic and optical images based on the cross-correlation of the image. We propose a matching algorithm for underwater optical and acoustic images.

The algorithm consists of training and matching. In the first step, we need to extract the spatial features of the preprocessed images, and use the spatial features of the images to train the correlation filter. In the second step, after completing the training process of the filter, the images are expanded to the Gaussian pyramid, and the correlation filter is used in each layer for detection. Finally, accurate matching results are obtained by fusing the multi-scale detection results.

Traditional image matching is generally based on image features. The accuracy of this kind of algorithm is greatly affected by the actual situation of underwater image edge blur, serious scattering, increased noise, etc. [30,31,32]. We designed a detector based on cross-correlation, which was used for image matching. It is not sensitive to fuzzy edges, incoherence, and the slight distortion of underwater images, and has a high tolerance for noise. Specifically, we used the minimum output sum of the squared error filter (MOSSE) for practical operation. The algorithm is robust to noise and does not depend on statistical properties, which means that it can better adapt to the scene of underwater images. In addition, the algorithm only needs a small number of training samples in the training process, and can even be trained using one picture. The methods are described as follows.

#### 3.4.1. Filter Training

In correlation filtering, the concept of cross-correlation is mainly used to express the similarity degree of two signals. The more similar the two targets, the greater the correlation value. Matching with the MOSSE filter is used to find a filter H to maximize its response on the target. The response value is calculated as follows:

(2) g=f⊗h

where g is the response output, f is the input image, and h is the filter template. To facilitate calculation, we transform the above formula into:

(3) $$\mathrm{FFT}\left(g\right)=\mathrm{FFT}\left(f\right)\cdot \mathrm{FFT}\left(H^{*}\right)$$

This can be expressed as the following formula:

(4) $$G=F\cdot H^{*}$$

Therefore, the filter can be solved according to the formula:

(5) $$H^{*}=\frac{G}{F}$$

In the process, we regard the region of the target object in the initial training sample image as the input image f, the corresponding response output g is generated by Gaussian function, and its peak position is at the center of f. A series of training samples are obtained by the projective transformation and affine transformation of the target region, and the operation can improve the robustness of the filter to rotation and deformation. The filter is initialized with multiple sets of training images:

(6) $$H_{i}^{*}=\frac{G_{i}}{F_{i}}$$

To solve the above filter template, the least-square method is introduced to find the actual parameters of the filter by minimizing the square of the error. In the actual training process, the variance between the actual output convolution and the desired output convolution is minimized to get the appropriate filter. Therefore, the training process needs to solve the error function of the minimum output sum of squared error filter, as follows:

(7) $$\underset{H^{*}}{\min}\sum_{i}{\left|F_{i}⊗H^{*}-G_{i}\right|}^{2}$$

Using the steepest descent method, the training process can converge within a few iterations, and the filter template $H^{*}$ can be obtained.

#### 3.4.2. Target Matching

After the filter template training of MOSSE, the acoustic and optical image matching process can be carried out [33]. Due to the differences in the image angles and the imaging principle between underwater optical images and acoustic images, there may be obvious differences of angle and size between two images of the same object. Therefore, we use the Gaussian scale-space to perform multi-scale matching on the images to be detected, and optimize the matching results of correlation filtering. This improvement makes the algorithm insensitive to angle and scale differences. The algorithm flow is as follows.

In the first step, we expand the images to be matched into a Gaussian pyramid. In other words, through Gaussian blur and down-sampling of the original images, a series of small to large images to be matched are obtained. The Gaussian scale-space can retain the main regional features of the original images, although the details are ignored to some extent. However, since the MOSSE filter is based on region correlation rather than the feature, its matching accuracy is less affected.

In the second step, each layer of the Gaussian pyramid is transformed by FFT. The MOSSE filter is applied to the transformed images to obtain the matching result. According to the correlation value between the trained filter $H^{*}$ and the image F to be matched, the peak value of the response images is the matching target. The matching process is carried out from the upper layer of the Gaussian pyramid to the lower layer. The high-level images can quickly search for candidate regions after multiple down-sampling. Then the corresponding region of the next layer continues to match. Once the result of the matching probability is higher than the specified threshold, the matching ends. If the volume of the target object is large, the matching is successful at the high level of the pyramid, and the operation time is saved. If the target volume is small, the matching accuracy can be guaranteed through layer-by-layer matching.

Using the minimum output sum of squared error filter in combination with the Gaussian scale space, the problems of object matching with large scale and angle difference between underwater optical images and underwater acoustic images are solved.

## 4. Results

In this section, we experimentally validate our proposed method on 10 sets of underwater detection data, each of which contains five pairs of corresponding acoustic and optical images (i.e., 50 sonar images and 50 optical images). If the target object is in the sound shadow, it will completely change the appearance of the object in the sonar image, which will affect the matching effect. To avoid the influence of the sound shadow on sonar data collection, obstacles between the sonar and target object should be avoided as much as possible in the process to ensure that the object is in the area that the sound wave can reach directly. First, we carried out random experiments using the actual matching success rate to prove the effectiveness of the proposed method. Then the importance of the iterative enhancement parameters and the generated filter template is analyzed in detail. Finally, our method is evaluated on these 10 datasets, and the matching performance of underwater images is compared with other methods.

### 4.1. Dataset

Two types of underwater images were required for the experiment. We used a Tritech-Micron type forward-looking sonar and high-definition monocular camera with waterproof treatment to for data collection. The data collection process was carried out in an experimental pool. We used a steel frame structure to fix the camera and sonar to keep it in a fixed position relative to the target object, and collected the acoustic and optical images in the same calibration position of the steel frame. Sediment was laid at the bottom of the experimental site to simulate the marine environment. The target objects were mainly shellfish, sea urchins, stones, and other common underwater objects. The temperature of the experimental site was 17 °C ± 2 °C. The salinity was set at 35‰ according to the marine environment. The optical image acquisition was carried out under normal illumination, and an underwater lamp with luminous flux of 600 LM and color temperature of 3000 K was used to provide additional illumination. The objects to be matched in each group were different types of underwater targets, which have more diversity and are closer to those of a real-world underwater scene. Therefore, we used this dataset to verify the proposed matching system. According to the number of objects in the matching scene, the underwater data set is divided into two subsets: complex and simple. The images in the complex set contain more than three objects, while the images in the simple set contain only one or two objects. Compared with simple subsets, complex subsets contain many more complex scenes. As expected, it is challenging to achieve excellent detection performance on the complex subset.

### 4.2. Implementation Details

In the iterative enhancement, we set the initial color deviation retention parameter θ = 0.5 in the automatic white balance algorithm, the ambient light retention parameter α = 0.1 in the dark channel priority algorithm, and the morphological filter constraint e = 15. During the enhancement process, we set the threshold for the end of enhancement to 5.6. In each matching process, the object with characteristic shape and material was selected as the target object (such as starfish or shellfish), and the volume of the target object was not less than 0.05 m³. The steel frame structure was used to ensure that the distance between the camera or sonar and the target object was 60 cm, and the depth of the object was 120 cm from the water surface. The shooting process of each group of images was carried out at the same place and the same illumination was used. Five enhanced results were taken as an example, and these experimental results are shown in Table 1. In the five examples, when the initial parameters are used, the values of PSNR and SSIM range from 0.0073 to 0.5277, and from 3.3679 to 10.0480. According to the calculation formula of SI, the SI value of most initial images does not reach the end threshold, so we need to continue the iteration. After iterating, the SI value finally exceeds the specified threshold value, and the enhancement ends.

**Figure 3:**
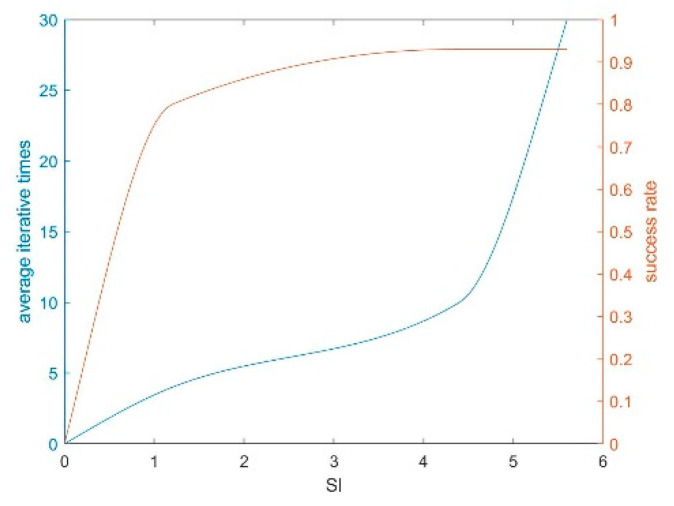
The trend of success rate and average iterative times on similarity index (SI).

In the actual experiment, the number of iterations and the effect are mainly determined by the SI threshold. As can be seen in Figure 3, the enhancement end threshold and the final matching success rate are positively correlated within a certain range, but the high threshold will increase the number of iterations and may lead to infinite iterations due to the difference of the image itself.

**Figure 4:**
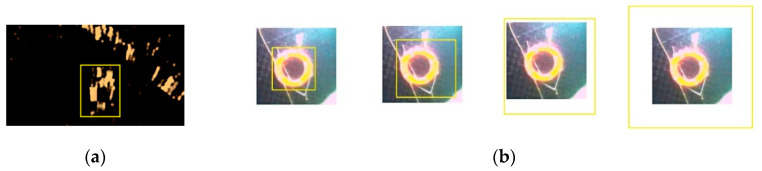
The process of multiscale matching. In subfigure (**a**), we select the target object in the acoustic image, and subfigure (**b**) shows its matching process in each layer of Gaussian scale-space.

In the matching experiment, we took a classical match in a simple set as an example. We calibrated each group of acoustic images and optical images manually, and took the result of sonar image manual calibration as the input image (Figure 4a). A series of training samples were obtained by the transmission and affine transformation of the input image, and the operation could improve the robustness of the filter to rotation and deformation. Gaussian expansion was applied to the optical images, and the filter template was used to match each layer of the extended images. The matching effect is shown in Figure 4b.

### 4.3. Comparison with Other Methods

**Figure 5:**
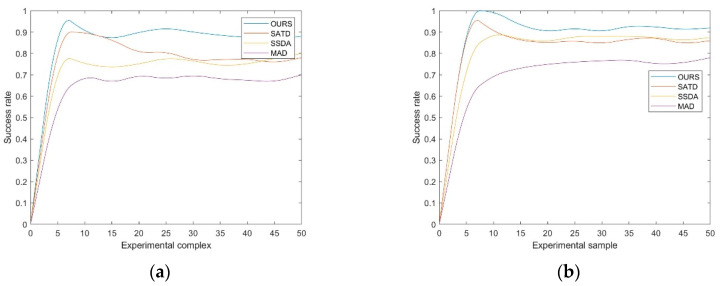
For the complex and simple sets, we compare our method with several classical methods. The subfigures (**a**) shows the performance of each method in complex sets, and (**b**) show the result in sample sets.

After several experiments, we randomly selected the data and compared our method with other common image matching methods on the average experimental results of 50 matching experiments [34,35]. Figure 5 shows the performance of each method in underwater image matching. From Figure 5, we can see that our method achieves the highest performance (88%) on the complex set, which is more than 1% higher than the performance of the other methods. Compared with these methods, our performance improvement mainly comes from the following aspects: (1) the use of various enhancement algorithms in the preprocessing process reduces the noise and other interference factors, and the detailed information is fully exposed; (2) the method based on correlation is not sensitive to the angle change; (3) the multi-scale images constructed by Gaussian blur and down-sampling are used to solve the problem of different sizes. In addition, we also obtained the highest performance (92%) on the simple subset, which is 5% higher than that of other matches. This is because, compared with the work in the complex set, the simple set has fewer interference objects, reducing the probability of identifying the interference objects as the target objects.

**Figure 6:**
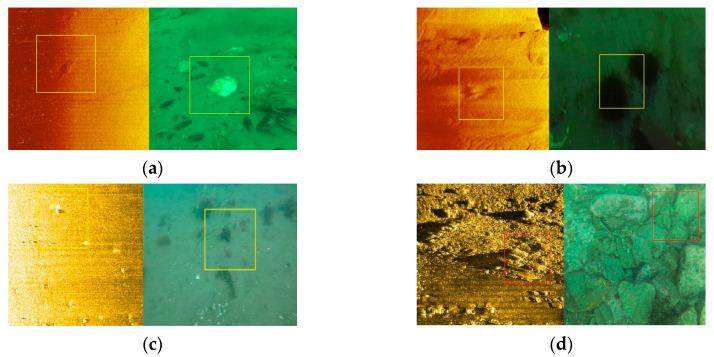
The results of matching. The subfigures (**a**), (**b**), (**c**) and (**d**) in the Figure 6 contains different types of objects, in which (**a**), (**b**), (**c**) matcheing successfully and (**d**) failed.

In Figure 6, we show some of the test results generated by our proposed method. It can be seen that our matching method successfully matches almost all of the targets, although some of them are small and fuzzy. In the matching results, we use the yellow box to display the matching result (that is, a, b, c) when the match is successful, and the red box is used to display the matching result (i.e., d) when the match fails. In image a, the target objects are shellfish, and in b and c they are sea urchins, which have obvious shape features. The matching target in image d is stone; its shape feature is not obvious enough, and there are many similar objects in the image, which is the main reason for the failure of matching. However, Figure 6 also shows some failure cases, which were mainly caused by two aspects: 1. Poor image quality causes the object to be too blurred in the image. 2. The object is in an acoustic shadow due to object occlusion.

## 5. Discussion

Compared with other matching methods, our method improves underwater image matching in terms of the following aspects. Compared with other feature-based matching algorithms, the filter based on regional correlation has the greatest advantage of being insensitive to the angle and scale of the images to be matched. This method can eliminate the change in the imaging and environment caused by the change of the angle of view used to take the image. Compared with matching using original images, the enhanced images reduce the noise of various acousto-optic sensors, increasing the proportion of effective information and the signal-to-noise ratio in the images, which helps improve matching accuracy. When the morphological filter is used to suppress noise, the edge of the target is smoothed, which helps reduce the impact of underwater object imaging imperfection and deformation. Finally, Gaussian multi-scale images are used to optimize the matching results and reduce the error caused by scale. Furthermore, if the volume of the target object is large, the matching is successful at a high level, which reduces the operation time. If the volume of the target is small, the matching accuracy can be guaranteed through layer-by-layer matching. The experimental results show that our method can accurately perform acoustic optical image matching.

## Figures and Tables

**Table 1:** Five enhanced results.

Initial SSIM	Initial PSNR	Initial SI	Final SSIM	Final PSNR	Final SI
0.3773	5.2023	5.5228	0.3670	11.0787	7.1770
0.2636	8.0049	5.1693	0.2772	10.8896	6.1775
0.0073	3.3679	1.0868	0.4411	3.3679	5.6423
0.2214	10.0480	5.3393	0.5490	7.5381	8.0262
0.5277	6.3500	7.4460	0.5277	6.3500	7.4460

Enhanced results.
